# Combined Intrathoracic and Intraspinal Approach to a Neurogenic Dumbbell Tumour

**DOI:** 10.7759/cureus.69248

**Published:** 2024-09-12

**Authors:** Juin Yi Ng, Khairul Anwar B Abdul Rahman, Jazmine Mohd Ramzisham, Hairulfaizi B Haron, Mohd Ramzisham B Abdul Rahman, Nur Ayub Md Ali

**Affiliations:** 1 Cardiothoracic Surgery, Hospital Canselor Tuanku Muhriz, Kuala Lumpur, MYS; 2 Surgery, Faculty of Medicine, The National University of Malaysia, Kuala Lumpur, MYS; 3 Cardiothoracic Surgery, The Royal College of Surgeons in Ireland (RCSI) and University College Dublin (UCD) Malaysia Campus, George Town, MYS; 4 Cardiothoracic Surgery, Faculty of Medicine, The National University of Malaysia, Kuala Lumpur, MYS

**Keywords:** neurilemmoma, neuroma, neurosurgery, schwannoma, thoracic surgery, video-assisted thoracoscopic surgery

## Abstract

A neurogenic dumbbell tumour is a rare occurrence in which the tumour has an extension through an intervertebral foramen, acquiring an hourglass shape. Surgical strategies to resect these tumours are rapidly evolving, and there is no definite consensus on the approach, stages, and plan of surgery. Here, we present a case report on a dumbbell tumour that was successfully resected via laminectomy and video-assisted thoracoscopic surgery (VATS) approach. A 53-year-old lady had an incidental finding of a mediastinal mass from chest radiography. Computed tomography (CT) and magnetic resonance imaging (MRI) studies showed a neurogenic mass with extension into the posterior mediastinum via the T1/2 neural foramina. A multi-disciplinary operation was planned for the patient, starting with neurosurgery resecting the intraspinal portion via laminectomy. The posterior mediastinal portion of the mass was resected via the right VATS approach and was successfully resected entirely. Her operation was complicated with a cerebrospinal fluid (CSF) leakage, requiring a revisit surgery. She was subsequently discharged well. Histopathology examination of the resected mass confirmed the diagnosis of schwannoma. Surgical resection of a dumbbell tumour is challenging because it involves both the thoracic and neurosurgical fields. There is currently no consensus on the best way to approach a tumour. Multiple articles have discussed various approaches, such as single-stage versus two-stage surgery, VATS versus open incisions, and the plan or sequence of the surgery. Dumbbell tumours need to be assessed on a case-by-case basis, and a multidisciplinary approach involving both neurosurgery and cardiothoracic surgery in deciding the best surgical approach could ensure a successful resection.

## Introduction

A neurogenic dumbbell tumour is a rare occurrence in which the tumour has an extension through an intervertebral foramen, acquiring an hourglass shape [1]. Although 90% of these tumours are non-malignant, the majority of the patients present with neurologic symptoms due to spinal cord compression [1]. The name dumbbell tumour does not necessarily refer to the dumbbell shape. Rather, it is a conceptual term where the tumour connects two or more separate regions, such as intradural, epidural, and paravertebral spaces [2]. Surgical strategies to resect these tumours are rapidly evolving, with no definite consensus on the approach, stages, and plan of surgery. Due to their varied locations, dumbbell tumours present with varying clinical symptoms and pathological characteristics, requiring different surgical treatments [2]. Here, we present a case report on a dumbbell tumour that was successfully resected via laminectomy along with a video-assisted thoracoscopic surgery (VATS) approach.

## Case presentation

**Figure 1:**
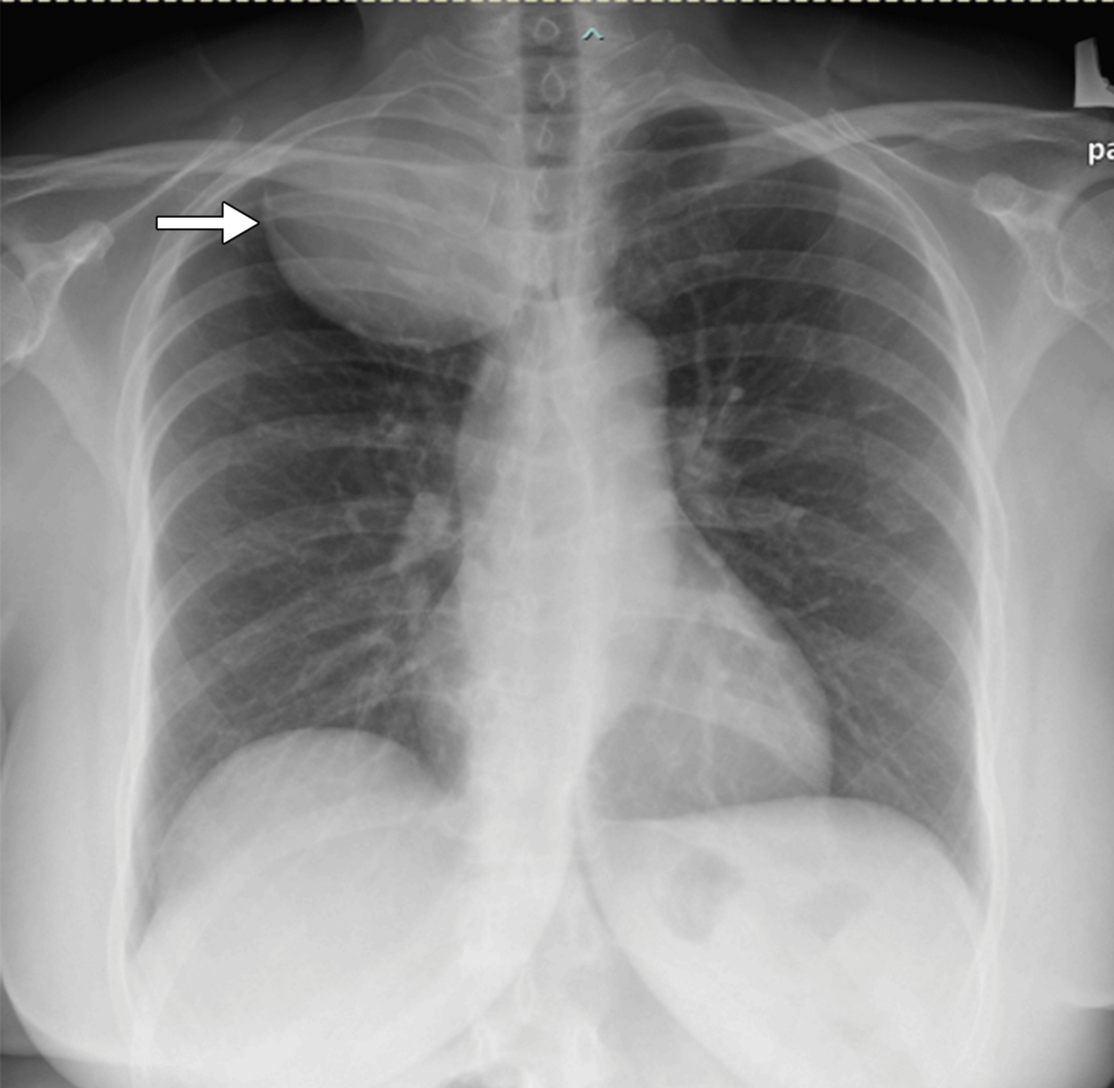
Chest radiography showing the intrathoracic portion of the dumbbell tumour (white arrow).

**Figure 2:**
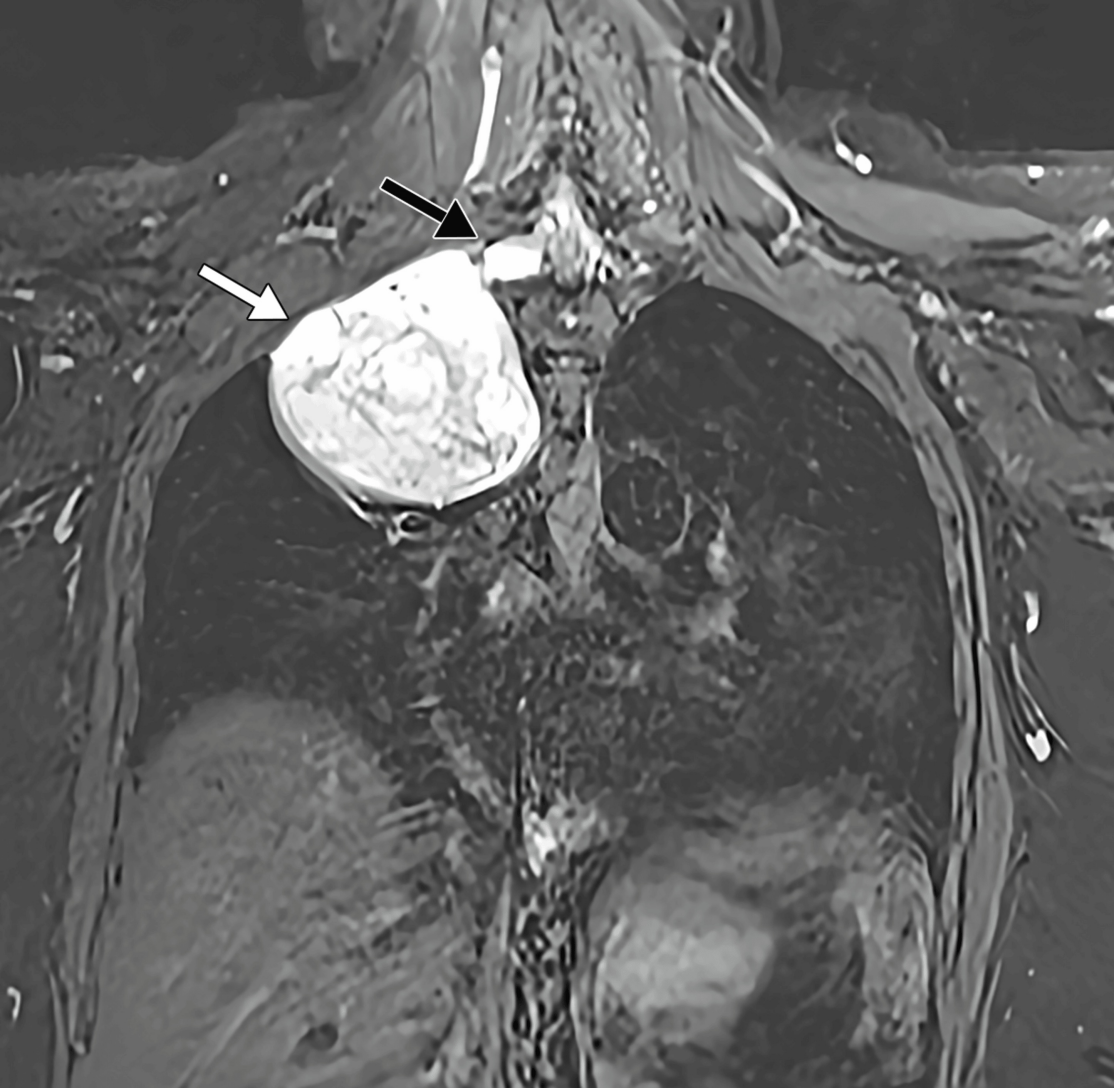
MRI image showing the dumbbell tumour (white arrow) with a smaller spinal portion of the tumour (black arrow). MRI: magnetic resonance imaging.

A 53-year-old lady had an incidental finding of a right upper mediastinal mass from chest radiography during a visit to the local clinic for an upper respiratory tract infection. A well-circumscribed mass with a smooth margin was seen in the right upper thorax, which crosses the midline (Figure 1). The patient was otherwise asymptomatic and had no neurological deficit. Computed tomography (CT) and magnetic resonance imaging (MRI) studies of the thorax confirmed a large intrathoracic, well-defined, heterogeneously enhancing solid cystic mass measuring 6.8 cm × 7.8 cm × 7.0 cm (antero-posterior × width × cranio-caudal). There is a continuation of the mass to the right T1/2 neural foramen, causing the widening of the T1/2 neural foramina and bony remodelling (Figure 2). It is causing minimal mass effect on the right-sided trachea with no significant narrowing.

**Figure 3:**
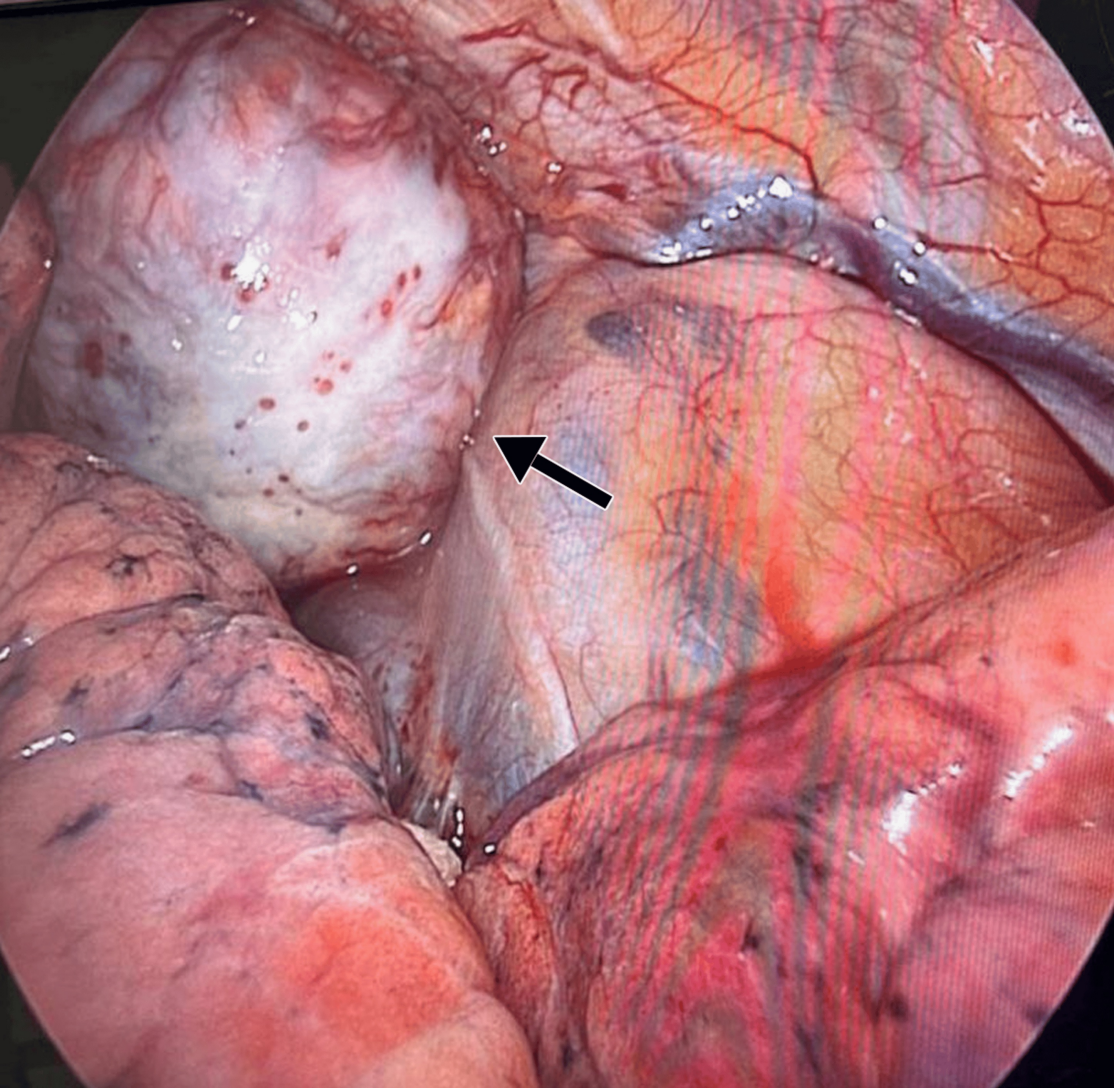
Intraoperative view via VATS of the intrathoracic portion of the tumour (black arrow). VATS: video-assisted thoracoscopic surgery.

A multi-disciplinary operation was planned for the patient, involving both neurosurgery and cardiothoracic surgery. Neurosurgery started by performing a costovertebral partial laminectomy of the T1 and T2. Intraoperative findings confirmed the tumour arising from the T1 and T2 nerve roots. The posterior mediastinal portion of the mass was resected via the right VATS approach and was successfully resected entirely. Intraoperatively, the tumour was seen over the right apex, pedunculated (Figure 3).

**Figure 4:**
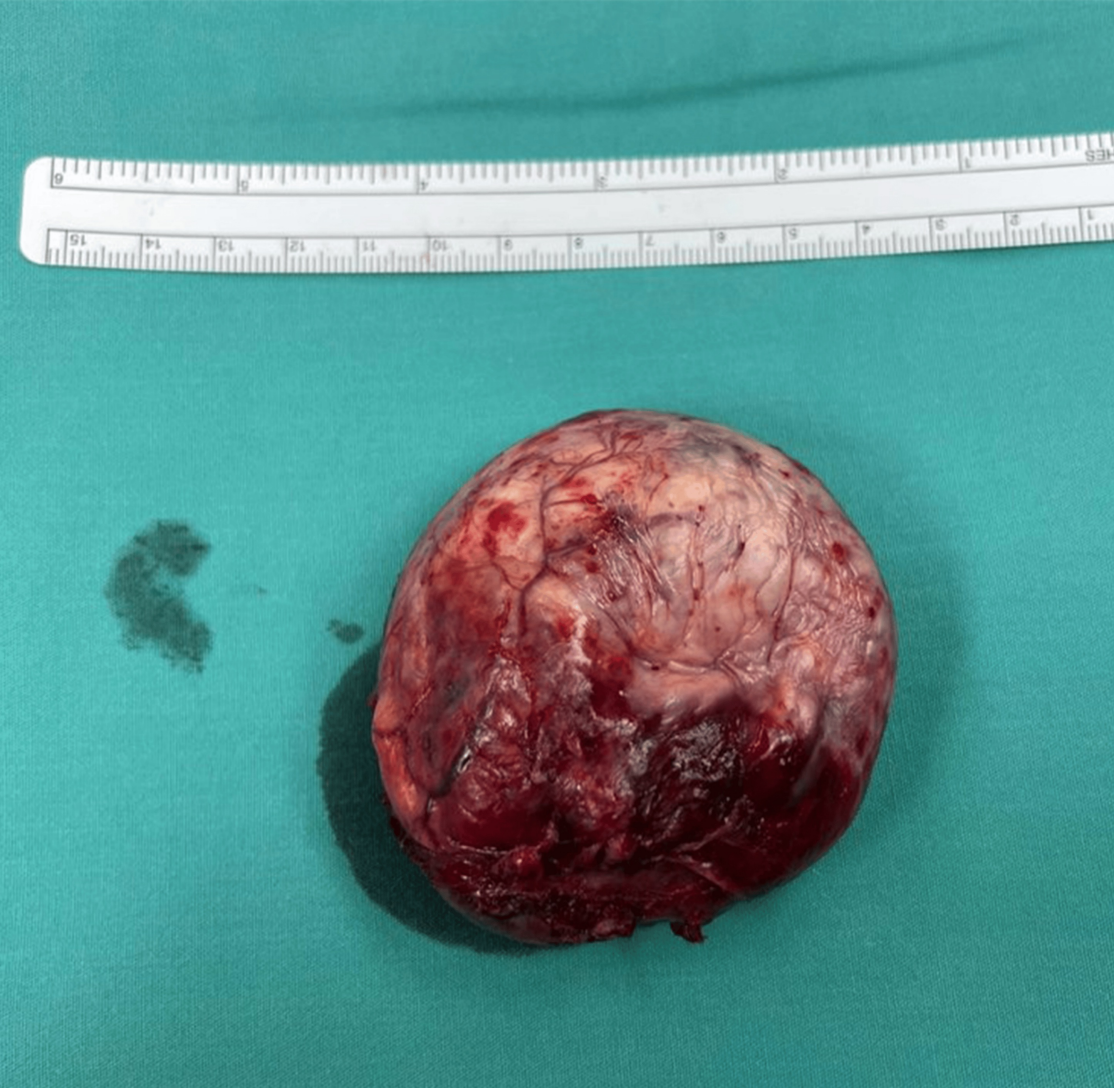
The resected extradural portion of the dumbbell tumour.

Postoperatively, the patient was unfortunately complicated with a cerebrospinal fluid (CSF) leakage, requiring a revisit surgery. The CSF leak was identified as originating from the T1/2 neural foramina and was sealed off with 5/0 polypropylene sutures with no further complications. She was subsequently discharged well. Histopathology examination of the resected mass confirmed the diagnosis of schwannoma or neurilemmoma.

## Discussion

Tumours arising from the nervous system and penetrating through the intervertebral foramen into the spinal canal are called dumbbell or hourglass tumours. The majority of these tumours are benign neurogenic tumours. They are typically composed of a bigger intrathoracic component and a smaller intraspinal portion. The benign tumours originated mostly from neuron sheath (68%), parasympathetic ganglion (30%), or paraganglionic cells (2%) [3]. These tumours were first described by Weber in 1856 and were named dumbbell tumours by Love and Dodge in 1952 [4].

The main treatment of choice for these tumours is surgical excision. It mainly involves the thoracic and neurosurgical fields. The resection of dumbbell tumours requires careful planning that combines neurosurgical and thoracic procedures as well as intra-thoracic manipulation. There have been multiple articles discussing the various approaches, such as single-stage versus two-stage surgery, VATS versus thoracotomy, and the order of the surgery. The earlier surgical strategy to remove the tumour was a two-staged surgery where an intrathoracic and intraspinal lesion was resected in two separate operations. In the 1970s came the one-stage removal of the dumbbell tumour, which is done via posterior laminectomy followed by posterolateral thoracotomy [1]. Following the development of VATS, then came the combined single-stage laminectomy-thoracoscopic removal of dumbbell tumours [5-7].

Single-stage surgery is favoured over two-stage surgery due to reduced complications [8]. Intra-foraminal resection of a tumour first in a two-staged surgery may produce neurological complications secondary to epidural haematoma or pseudomeningocele [1]. On the other hand, removal of the intrathoracic portion of the tumour only may cause spinal cord compression due to oedema of the residual component or haematoma [1,8].

A study by Li et al. showed that single posterior surgery versus combined laminectomy and thoracoscopic surgery were both effective for excision of the dumbbell tumour, with no tumour recurrence in either group. However, average operative duration, blood loss, hospitalisation, and rate of complications (e.g., temporary intercostal neuralgia, superficial wound infection, pulmonary atelectasis, and pneumonia) were significantly lower in the posterior approach [9].

With the advent of robotic surgery, there has been reported a single-staged combined spinal and robotic-assisted thoracic approach. The advantages include clear visualisation and no repositioning required, which in turn reduces the anaesthesia time [10]. Although some studies have reported intra-thoracic manipulation using robot-assisted surgery for improved safety and minimal invasiveness, neurosurgical procedures of hybrid surgery have been mainly performed using laminectomy. Hybrid spinal endoscopy and robot-assisted thoracoscopic surgery for dumbbell tumours is a procedure that is likely to become more prevalent with the advantage of localised destruction of dorsal bone structures and the flexibility of the robotic arm. A hybrid operation of spinal endoscopy and robot-assisted intra-thoracic surgery performed for dumbbell-shaped tumours provides a less invasive and safer procedure [11].

Dumbbell tumours are associated with higher rates of CSF leakage compared to non-dumbbell spinal nerve sheath tumours. Complication rates increase as the size of the tumour increases. This is caused by the dural defect in intradural-extradural tumours. Strategies to reduce the risk of CSF leak include usage of autologous fascia along with dural graft and fibrin glue; using non-penetrating vascular clips instead of needle and thread during suturing of dura; and application of autologous abdominal fat graft and fibrin glue [12]. Most cases of CSF leak are treated conservatively with antibiotics [13]; however, prolonged CSF leakage can lead to complications such as meningitis, arachnoiditis, delayed wound healing, and complications of intracranial hypotension (e.g., intracranial haemorrhage and cranial nerve palsy) [14].

## Conclusions

Dumbbell tumours need to be assessed on a case-by-case basis, and a multidisciplinary approach involving neurosurgery in deciding the best surgical approach could ensure a successful and complete resection.

Surgical resection of a dumbbell tumour is challenging because it involves both the thoracic and neurosurgical fields. There is currently no consensus on a unified way to approach the dumbbell tumours. The decision still depends on the individual surgeon and should be tailored to the patient and tumour characteristics. With the rapid evolution of surgical approaches such as robotic surgeries and the combined approach of spinal endoscopy and robot-assisted intrathoracic surgery, new approaches to resecting dumbbell tumours are showing promising results.
